# Comparing large-volume band ligators and cyanoacrylate injection for gastric variceal eradication: A prospective study

**DOI:** 10.1097/MD.0000000000031939

**Published:** 2022-11-18

**Authors:** Ding Shi, Jianping Liu

**Affiliations:** a Department of Gastroenterology, Ningbo No. 2 Hospital, Ningbo, China.

**Keywords:** endoscopic band ligation, endoscopic injection, gastric varices, tissue adhesives

## Abstract

The role of endoscopy in the ligation of gastric varices (GV) remains controversial. This study aimed to evaluate the efficacy of endoscopic band ligation (EBL) using large-volume ligators for the management of non-bleeding GV in patients with cirrhosis.

**Methods:**

One hundred fifty-eight patients with non-bleeding GV due to cirrhosis were divided randomly into 2 groups: the EBL group and the endoscopic variceal obturation (EVO) group. The EBL group underwent EBL with large-volume ligators and the EVO group underwent tissue glue injection for the treatment of GV. Follow-up endoscopy was performed 3 to 4 weeks after endoscopic treatment. Patients were followed up for ≥6 months after treatment. Eradication, complication, and recurrence rates were evaluated and compared between groups.

**Results:**

The type and size of GV were similar in both groups. No significant difference was found in the mean number of treatment sessions or eradication and recurrence rates after 6 months. Ulcer bleeding occurred in 2 EBL patients (2.50%) after ligation, whereas 8 EVO patients (10.25%) experienced bleeding due to glue extrusion. The bleeding rate after endoscopic treatment significantly differed between the groups. In the EVO group, 1 patient developed renal embolism after injection and 2 patients developed sepsis. The prevalence of postoperative fever was significantly higher in the EVO group than in the EBL group.

**Conclusion subsections:**

Large-volume band ligators have similar efficacy to tissue glue for eradicating GV, however, the former is safer. Nevertheless, multicenter studies are needed to further confirm these results.

## 1. Introduction

GV can be divided into the following 4 types according to Sarin’s classification system^[1–3]^:

1. gastroesophageal varices type 1 (GOV1), which are esophageal varices (EV) extending across the gastroesophageal junction to the lesser curvature of the stomach;
2. gastroesophageal varices type 2 (GOV2), which are EV extending across the gastroesophageal junction to the greater curvature of the stomach;
3. isolated gastric varices type 1 (IGV1), which are varices in the fundus of the stomach but not the esophagus; and
4. isolated gastric varices type 2 (IGV2), which are varices in the stomach outside of the cardio-fundal region or duodenal bulb.

Although the incidence of GV is only 20% in patients with cirrhosis, GV are associated with a higher risk of hemorrhage and mortality.^[4]^ The mortality rate is reportedly 20% within 6 weeks.^[5]^ The endoscopic treatment of GV is challenging because of their deep location and requires retroflexion of the endoscope for observation. Furthermore, GV are often accompanied by gastrorenal shunts.^[6,7]^

EVO is 1 method used to prevent GV bleeding. Several previous studies have compared the efficacy of EVO with the use of beta-blockers to prevent bleeding from GV and demonstrated the superior efficacy of EVO.^[8–10]^ However, tissue injection may lead to systemic embolism, which is associated with patient morbidity and mortality.^[11–14]^ Therefore, de Franchis et al^[10]^ suggested that the risk/ benefit ratio of tissue glue for prevention of gastric variceal bleeding should be evaluated before making recommendations. In addition, endoscopic ultrasound-guided injection can help prevent the occurrence of such adverse events; however, this method is technically challenging and requires complex equipment.^[15,16]^ As such, there is a clinical need to develop a simple strategy for the treatment of GV. EBL is a simple, safe, and effective method that is commonly used for the treatment of EV.^[17,18]^ Endoscopic ligation of GV was first reported in the early 1990s^[19–21]^; however, this method has not been widely accepted because of limitations in the devices used for ligation and because the indications for GV ligation are greatly limited.^[20–25]^ Moreover, in previous studies, it was found that detachable snares and nylon loops could only be used to ligate protuberant nodular or tuberous GV, whereas band ligators could only be used to ligate small- to moderate-sized GV due to their small volume. At present, there is no report comparing tissue glue and ligation in gastric variceal eradication.

We hypothesized that a large-volume band ligator could overcome the limitations of the detachable snares and small-volume ligators, and would be superior to endoscopic injection in the treatment of GV. Thus, we aimed to compare the efficacy and safety of EBL with large-volume band ligators to that of EVO in gastric variceal eradication, which, to our knowledge, has not been investigated previously.

## 2. Methods

### 2.1. Patient selection

This prospective, randomized controlled trial (Chinese Clinical Trial Registry, ChiCTR, No. ChiCTR1900027588) enrolled 158 patients (30–78 years) with GV due to cirrhosis who presented to our institute between March 2018 and November 2020. All patients in this study were diagnosed with GV (with or without EV) by endoscopy. The indication for treatment was primary prophylaxis in patients without a history of bleeding, whereas secondary prophylaxis was performed in patients with a history of bleeding, but without endoscopic treatment. Patients with a history of endoscopic treatment (endoscopic injection or band ligation), active esophageal or gastric variceal bleeding, or hepatocellular carcinoma were excluded.

Patients with confirmed GV were randomized using SPSS (SPSS version 22.0. SPSS Inc., Chicago, IL). The patients were randomly assigned to 1 of 2 groups (EBL and EVO groups; 1:1 ratio) using numbered envelopes containing the assigned endoscopic treatment. All patients underwent upper gastrointestinal endoscopy, and the following data were recorded: size and grade of GV (as mentioned above), presence and type of EV and portal hypertensive gastropathy.

GV form and size were classified as follows: F1 (small or mild, tortuous winding varices), F2 (medium or moderate, nodular-shaped varices), and F3 (large or severe, tumorous huge varices), according to the system suggested by Hashizume et al,^[1]^ Abby et al,^[26]^ and the American Association for Study of Liver Diseases 2007.^[27]^

The study was conducted in accordance with the Declaration of Helsinki, and the research protocol was approved by the Ethics Committee of Ningbo No. 2 Hospital. All included patients provided written informed consent.

### 2.2. EBL group

**Figure 1.**
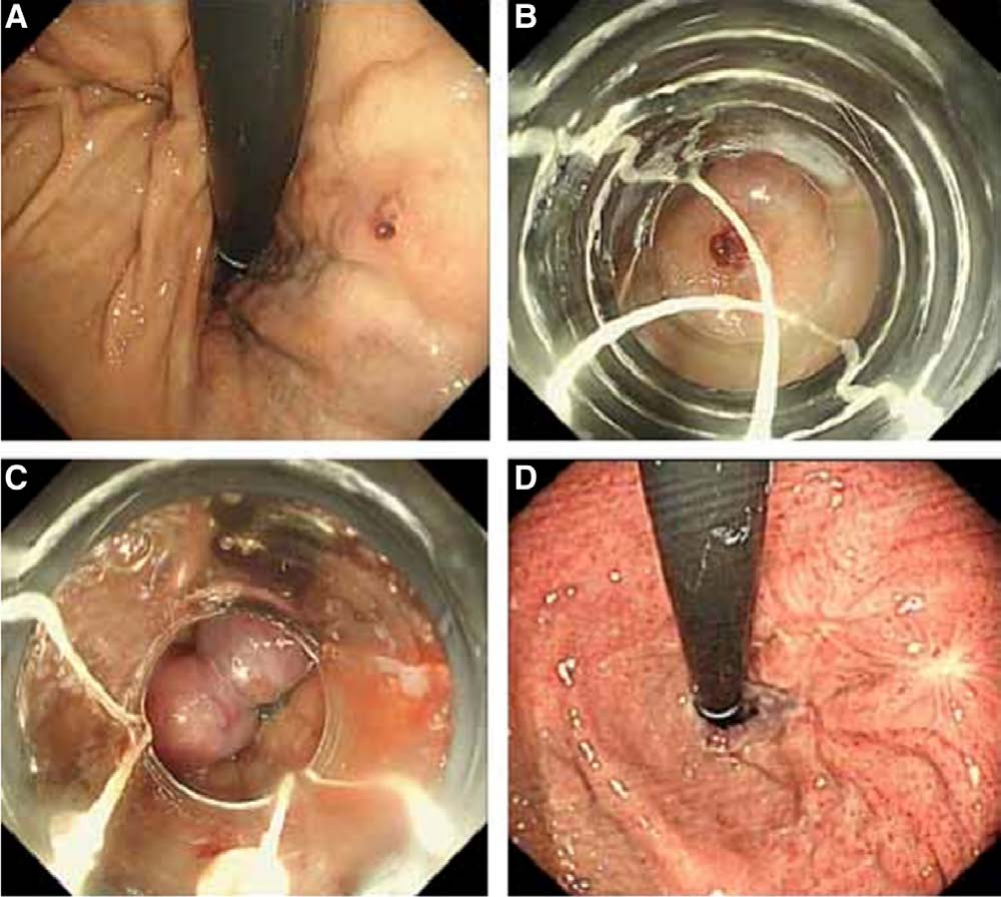
Ligation of GOV1. (A) Endoscopic observation revealed a red thrombus on the GOV1 GV. (B) Endoscopic view of the GV within the ligator cap. (C) After band ligation. (D) The GV had disappeared 4 weeks after EBL. EBL = endoscopic band ligation, GOV1 = gastroesophageal varices type 1, GV = gastric varices.

**Figure 2.**
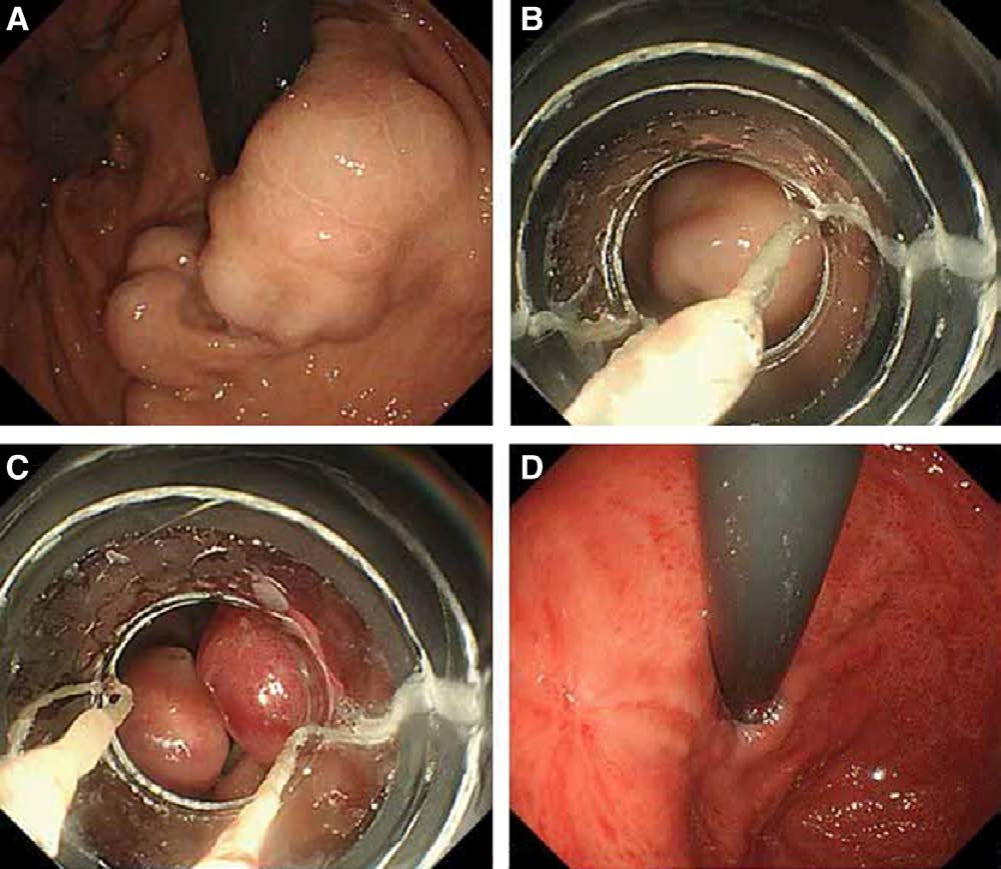
Ligation of GOV2. (A) Endoscopic observation revealed erosion after bleeding on the GOV2 GV. (B) Endoscopic view of the GV within the ligator cap. (C) After band ligation. (D) The GV had disappeared 4 weeks after EBL. EBL = endoscopic band ligation, GOV2 = gastroesophageal varices type 2, GV = gastric varices.

**Figure 3.**
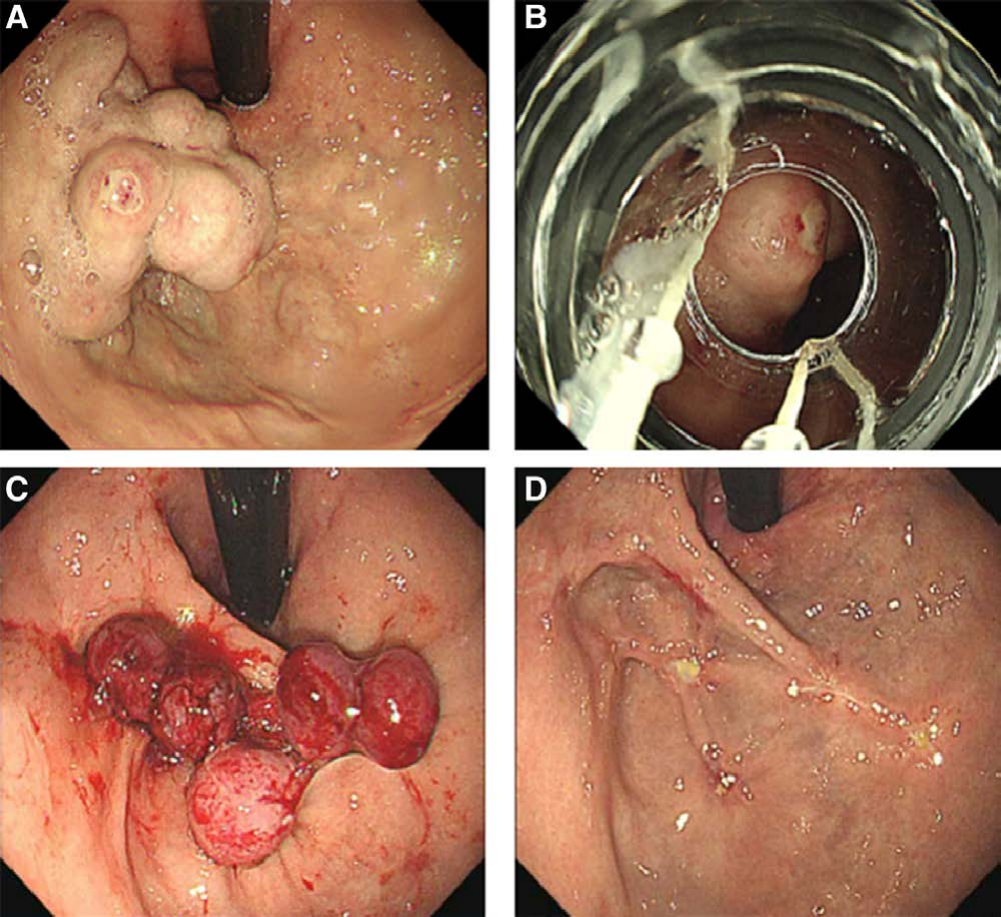
Ligation of IGV type 1. (A) Before band ligation. (B) Endoscopic view of GV within the ligator cap. (C) After band ligation. (D) The GV had disappeared 4 weeks after EBL. EBL = endoscopic band ligation, GV = gastric varices, IGV = isolated gastric varices.

**Figure 4.**
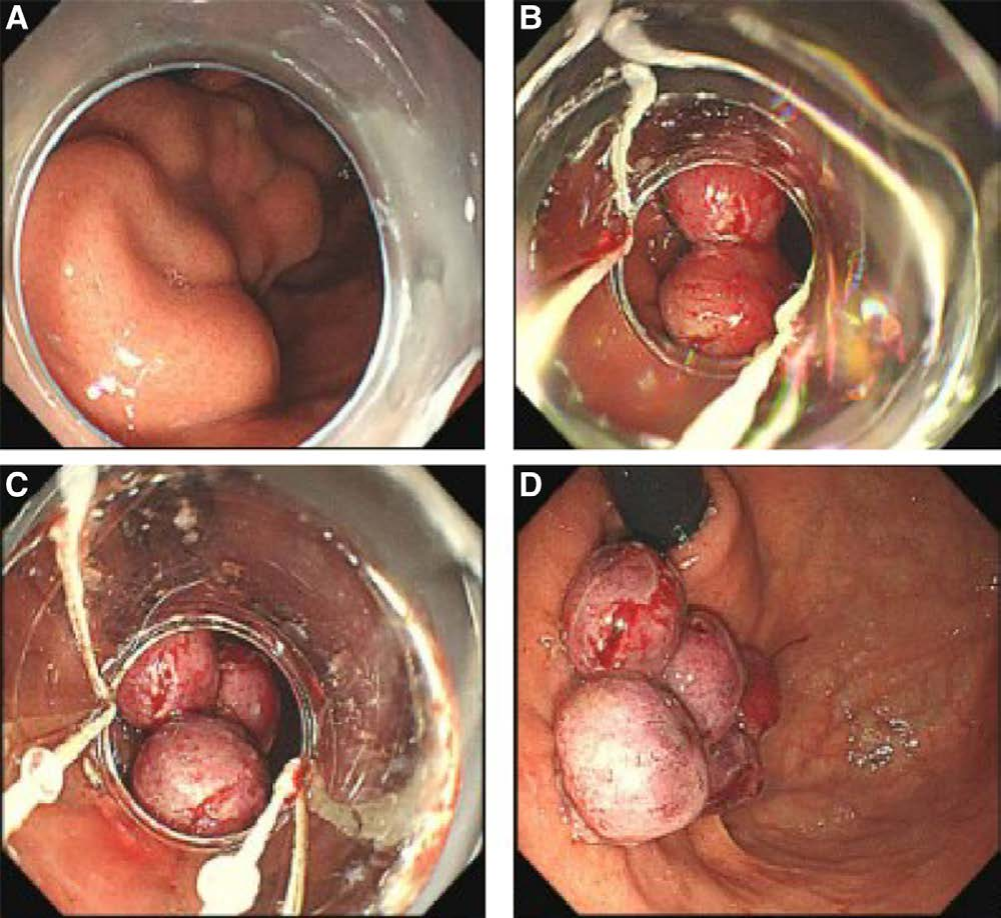
The ligation sequence of IGV type 1. (A) One end of the GV was ligated first. (B) Endoscopic observation of the second site, which was ligated clockwise. (C) Endoscopic observation of the third site, which was also ligated clockwise. (D) Endoscopic view after ligation. GV = gastric varices, IGV = isolated gastric varices.

Propofol (2/kg) was administered intravenously (iv) to induce anesthesia prior to EBL or EVO. The endoscope (9.9 mm; GIF-Q260J; Olympus Optical Co., Ltd., Tokyo, Japan) was fitted with the Saeed 10-Shooter Multi-Band Ligator (Wilson Cook, Winston-Salem, NC) and introduced into the fundus of the stomach. Subsequently, the endoscope was retroflexed and the GV (Fig. 1A, Fig. 2A, Fig. 3A and Fig. 4A) were ligated (Fig. 1B and C, Fig. 2B and C, Fig. 3B and C and Fig. 4B and D). Generally, the GV close to the cardia were ligated first, followed by those further from the cardia. For large IGV1 GV, 1 end or the side of the varices was ligated first, and then the adjacent varices were ligated in a clockwise or counterclockwise fashion (Fig. 4B and D). The distance between the 2 ligation points should be ≥1 cm; if necessary, the normal gastric mucosa adjacent to residual varices can be included. After ligation of the GV, ligation of the EV was performed immediately without replacing the band ligator. This procedure was performed at regular intervals of 3 to 4 weeks until the varices were eradicated.

### 2.3. EVO group

An endoscope (GIF-Q260J; Olympus Optical Co., Ltd., Tokyo, Japan), injection needle (Olympus NM-200L-423; Olympus), histoacryl blue (Beijing Fu’aile Technology Development Co., Ltd., Beijing, China), and lauromacrogol (Tianyu Pharmaceutical, Zhejiang, China) were used for EVO treatment employing the sandwich technique. Histoacryl glue (0.5 mL) with lauromacrogol (2 mL) was injected into the GV before and after tissue adhesive injection. The injection was stopped when the varices became engorged. Reinjection was performed in any residual GV while maintaining blood flow (depressive at palpation with the catheter) every 3 to 4 weeks until eradication. After EVO of GV, EBL was performed for concomitant EV.

To hasten the healing of ulcers induced by endoscopic therapy, a proton pump inhibitor and protective agent for the gastric mucosa were routinely administered to patients in both groups. After each endoscopic treatment session, the patients received esomeprazole 40 mg (AstraZeneca Pharmaceutical Co., Ltd, Wuxi, China) once daily via intravenous injection, and teprenone 50 mg (Weicai Pharmaceutical Co., Ltd, Suzhou, China) 3 times per day after meals.

### 2.4. Outcome measurements

The primary outcomes were gastric variceal eradication or recurrence rate and bleeding from GV after endoscopic treatment. The secondary outcomes were complications and adverse events related to endoscopic treatment, including sepsis, fever, and distant embolization.

### 2.5. Follow-up

Follow-up endoscopy was performed 3 to 4 weeks after endoscopic treatment. Patients were followed-up for ≥6 months following treatment completion.

In the EBL group, complete disappearance of dilated varices from the cardio-fundal region was defined as gastric variceal eradication (Fig. 1D, Fig. 2D, and Fig. 3D). Varices that were still visible in the cardia and fundus outside the ligation site after endoscopy were considered residual varices.

In the EVO group, gastric variceal eradication was defined as the complete obliteration or absence of GV, observed during follow-up endoscopy, that is, when all injected varices were whitish in color and hard on palpation with a catheter. Injected varices that were still elastic when touched with a catheter were not considered to be completely eradicated. Gastric variceal recurrence was defined as new GV identified by endoscopy after complete eradication.

Bleeding associated with endoscopic treatment was defined as bleeding due to glue extrusion or band slipping, which was treated using the same endoscopic method or a transjugular intrahepatic portosystemic shunt (TIPS). Ectopic embolism was defined as emboli in organs other than the gastroesophageal tract that could lead to serious clinical consequences.

### 2.6. Statistical analysis

Based on previous studies, the bleeding rate of ulcers after band ligation was predicted to be approximately 2% and the bleeding rate due to glue extrusion approximately 22%.^[23,28]^ The minimum required sample size for this study was 45 patients in each group. Statistical analyses were performed using SPSS for Windows (version 22.0. SPSS Inc., Chicago, IL). Continuous variables are expressed as the mean ± standard deviation and were compared using Student t-test for 2 independent samples. Categorical data are expressed using absolute frequencies and percentages and were compared with the Chi-square test or Fisher exact test. Gastric variceal recurrence and mortality rates were determined using the Kaplan–Meier method and compared between groups using the log-rank test. If the same method was used to treat gastric variceal bleeding after endoscopic treatment, it would be considered as the second treatment of the same method to count the eradication and recurrence rate. Otherwise, it was considered that this method did not achieve gastric variceal eradication. EV bleeding after endoscopic treatment was not included in this study. All tests were 2-tailed, the confidence interval was 95%, and a *P*-value of < .05 was considered to indicate statistical significance.

## 3. Results

Of the 162 patients enrolled, 158 (female/male 114/44; mean age 57.23 yrs, range 30–78 yrs) were included in the analysis. Four patients who refused endoscopic treatment were excluded. Participant flow is shown in Figure, Supplemental Digital Content 1, http://links.lww.com/MD/H984. Except for portal hypertensive gastropathy, there were no significant differences in demographic and clinical characteristics between patients in the EBL and EVO groups (Table 1). Furthermore, the types and size of GV were similar between the 2 groups (Table 1). Cirrhosis due to viral hepatitis was the most common underlying liver disease, followed by alcoholic liver disease. In this study, 25 patients were treated as primary prophylaxis and 133 patients were treated as secondary prophylaxis. No patients in either group had isolated gastric varices type 2 (IGV2) GV, and GOV1 and GOV2 GV accounted for 80.4% of cases in the 2 groups. The maximum diameter of IGV1 GV treated by EBL was 2.7 cm (Fig. 4A). As the patient distribution was randomized, the groups were not equally balanced for portal hypertensive gastropathy, which was more severe in the EBL group than in the EVO group (*P* = .019).

**Table 1:** Demographic and clinical characteristics.

Characteristics	EBL (n = 80)	EVO (n = 78)	*P*-value
Sex (male/female), n	56/24	58/20	0.333
Age (yrs), mean ± SD	57.30 ± 10.60	57.15 ± 10.04	0.755
Cause of cirrhosis, n			0.408
Alcoholic liver disease	8	13	
Chronic hepatitis B + C	62	60	
Autoimmune hepatitis	7	5	
Other	3	0	
Portal hypertensive gastropathy, n (%)	46 (57.50%)	31 (39.74%)	0.019
Ascites (present/absent), n (%)	47 (58.75%)	39 (50.00%)	0.173
Portal vein diameter (mm)	14.51 ± 2.69	14.51 ± 2.79	0.944
Child-Pugh class (A/B/C), n	15/45/20	17/52/9	0.096
GV type (GOV1/GOV2/IGV1), n	41/21/18	38/27/13	0.790
GV degree (F1/F2/F3), n	1/44/35	1/48/29	0.421
Size of GV	13.12 ± 2.35	13.21 ± 3.02	0.397
History of bleeding (present/absent), n (%)	69(85.00%)	64(82.05%)	0.307

EBL = endoscopic band ligation, EVO = endoscopic variceal obturation, GOV = gastroesophageal varices, GV = gastric varices, IGV = isolated gastric varices, SD = standard deviation.

The effects and complications of endoscopic treatment are presented in Table 2. The technical success rates of both groups were 100%. No significant difference in the mean number of treatment sessions was found between the groups (*P* = .346). At the 6-month follow-up, GV were eradicated in 75 patients in the EBL group and in 72 patients in the EVO group. No significant differences in the eradication (*P* = .482) or recurrence (*P* = .512) rates at 6 months of follow-up were observed between the groups. GV recurred in 3 and 2 patients in the EBL and EVO groups, respectively. In the EBL group, 2 patients (2.50%) experienced ulcer bleeding after ligation, and 8 patients (10.25%) experienced late bleeding due to glue extrusion in the EVO group. A significant difference in the rates of bleeding after endoscopic treatment was noted between the groups (*P* = .045). Among the 2 patients who experienced bleeding after EBL, 1 underwent repeat EBL. Among the 8 patients who experienced bleeding after EVO, 5 underwent repeat EVO. The other 4 were treated with transjugular intrahepatic portosystemic shunt (TIPS). Moreover, in the EVO group, 1 patient developed a renal embolism 3 days after injection and recovered after 2 weeks of conservative treatment, while 2 patients developed sepsis and recovered after effective antibiotic treatment with cefoperazone sodium/sulbactam sodium (Cefoperazone Sodium and Sulbactam Sodium for Injection) 2 g intravenously every 12 hours. The rate of postoperative fever was significantly higher in the EVO group than in the EBL group (*P* = .002). In the EBL and EVO groups, for gastroesophageal type varices, the mean numbers of treatment sessions to eradicate EV were 2.13 ± 0.63 and 2.14 ± 0.67, respectively, while the mean numbers of treatment sessions to eradicate GV were 1.43 ± 0.52 and 1.35 ± 0.53, respectively. In either group, the mean number of treatment sessions to eradicate EV was significantly higher than that to treat GV (EVL group, *P* = .015; EVO group, *P* = .014).

**Table 2:** Results of treatment and complications.

Characteristics	EBL (n = 80)	EVO (n = 78)	*P* value
Sessions to GV eradication, mean ± SD	1.43 ± 0.52	1.35 ± 0.53	.355
Eradication rate at 6 months’ follow-up, n (%)	75 (93.75%)	72 (92.31%)	.482
GV bleeding after endoscopic treatment, n (%)	2 (2.50%)	8 (10.25%)	.045
Sepsis, n (%)	0 (0.00%)	2 (2.56%)	.242
Fever, n (%)	9 (11.25%)	24 (30.77%)	.002
Distant embolization, n (%)	0 (0.00%)	1 (1.28%)	.494
Recurrence, n (%)	3 (3.75%)	2 (2.56%)	.512

EBL = endoscopic band ligation, EVO = endoscopic variceal obturation, GV = gastric varices, SD = standard deviation.

## 4. Discussion

In this study, we aimed to compare the effect of EBL with large-volume band ligators with that of EVO in the management of GV. Although the incidence of GV is lower than that of EV, gastric variceal bleeding is often more severe and may be life-threatening.^[29]^ Currently, no generally accepted therapeutic intervention for GV has been established. However, tissue adhesive injections have the largest evidence base and are currently the preferred method of treatment; ^[30]^ meanwhile, data regarding ligation in the treatment of GV is limited.

Our study compared the efficacy and safety of EBL with large-volume band ligators with that of EVO in the treatment of GV. Regarding GV types, GOV1 accounted for 50.00%, GOV2 for 30.38%, and IGV1 for 19.62% of all cases of GV in this study, which is comparable to previous studies.^[6]^ Moreover, in this study, the success rate of EBL in the treatment of GV was 93.75%, which was not statistically significant compared with that of EVO, and was markedly higher than that previously reported, while the GV recurrence rate was 3.75%, which was not statistically significant compared with that of EVO and was markedly lower than that previously reported.^[21,23]^ One possible explanation of these findings is that the ligators used in our study were large-volume ligators (Cook 10-Shooter Multi-Band Ligator), while the ligators used in previous studies were small-volume ligators (Cook 6-Shooter Multi-Band Ligator or pneumo-activated ligator).^[21–25]^ A large-volume ligator has a longer ligation cylinder, which can hold more varices and tissues, and as such, the depth of ligation is closer to the submucosa, thereby ligating the GV more thoroughly. Although large GV cannot be completely attracted and ligated at 1 time, the softness and deformability of varices makes them partially attracted into the transparent cap and ligated, and any residual varices can still be attracted and ligated again. The results of this study showed that EBL was a feasible method for the treatment of large GV.

Furthermore, the incidence of complications was significantly higher in the EVO group than that in the EBL group. In the EBL group, the rate of bleeding from ligation ulcers was 2.50%, which was markedly lower than that in the EVO group (10.25%). Based on previous studies, the prevalence of EBL-induced ulcer bleeding was 3.6 to 15%.^[5,31,32]^ However, other studies have suggested that ligation ulcers are not the most important risk factors for bleeding after EBL, as bleeding at ligation ulcers occurred in only 0.5% (1/205) of elective EBL cases.^[31]^ In our study, we aimed to perform elective EBL in patients with non-bleeding GV, and a strong proton pump inhibitor and protective agent for the gastric mucosa were used to hasten the healing of ulcers, thereby reducing the risk of ulcer bleeding. However, as glue discharge is an inevitable process, bleeding due to glue extrusion, i.e., bleeding caused by breakage during the removal of the glue, is sometimes unavoidable. In this study, the rate of bleeding due to glue extrusion in the EVO group was 10.25%, which was within the range reported previously (5.6–22%).^[28,33]^

In this study, 1 case of renal embolism and 2 cases of sepsis occurred in the EVO group but not the EBL group, and the EBL group also had a much lower incidence of postoperative fever than did the EVO group, suggesting that EBL had obvious advantages over EVO in avoiding these complications. So far, distant embolism is a congenital defect of tissue glue injection and cannot be completely avoided,^[34]^ which is precisely the advantage of EBL. Although the incidence of sepsis is extremely low (2.56%), the prevention of sepsis is challenging, as the injection needle can be contaminated with bacteria from the oral and gastrointestinal tracts.^[35]^ Transient bacteremia frequently occurs after endoscopic treatment procedures, such as sclerotherapy or tissue adhesive injections,^[36,37]^ and can result in sepsis.^[38]^ Approximately 90% of patients experience transient fever after the endoscopic injection of tissue adhesives,^[39]^ which could explain the significantly higher incidence of postoperative fever in the EVO group compared to that in the EBL group in our study.

Overall, our findings indicate that large-volume ligators avoid distal embolization and sepsis and reduce postoperative bleeding and fever; thus, these are safer than tissue glue injections in the treatment of GOV GV.

This study has some limitations. This was a single-center study and the follow-up period was short. Thus, large multicenter studies are needed to further confirm our findings.

In conclusion, the efficacy of EBL with large-volume ligators in the eradication of GV is similar to that of tissue glue injection; however, the former is safer. Thus, we recommend the use of large-volume band ligators for gastric variceal eradication to reduce the rate of complications.

## Acknowledgements

We would like to thank Yideji for the English-language editing of this article.

## Author contributions

DS designed the study. JL analyzed the data. DS and JL jointly interpreted the data and drafted the manuscript.

**Conceptualization**: Ding Shi.

**Data curation**: Jianping Liu.

**Formal analysis**: Ding Shi.

**Funding acquisition**: Ding Shi.

**Investigation**: Jianping Liu.

**Methodology**: Ding Shi.

**Writing – original draft**: Ding Shi.

**Writing – review & editing**: Jianping Liu.

All authors read, critically revised, and approved the final manuscript.

## Supplementary Material
