# Supplementary material for: Comparing large-volume band ligators and cyanoacrylate injection for gastric variceal eradication: A prospective study
Source: Medicine (Baltimore). 2022 Nov 18;101(46):e31939. doi: 10.1097/MD.0000000000031939 (PMC9678509; doi:10.1097/MD.0000000000031939)
Supplement: Supplementary file 1 [file medi-101-e31939-s001.pdf]

Comparing large-volume band ligators and cyanoacrylate injection for gastric variceal eradication: a prospective study

Ding Shi MD

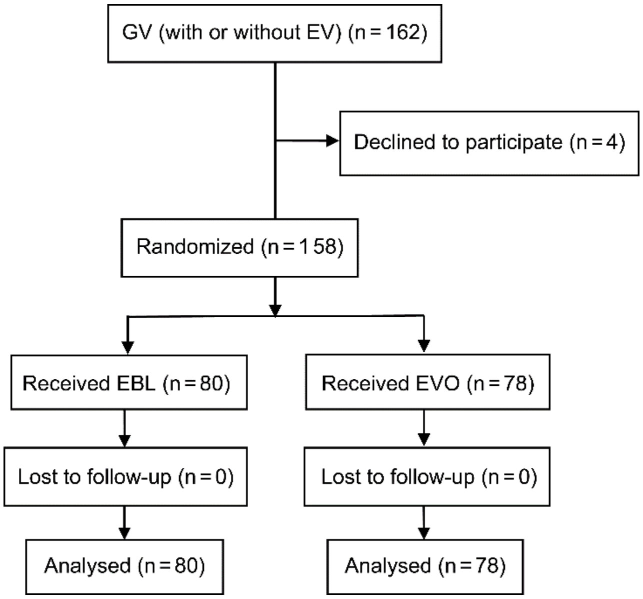

Supplemental Digital Content 1. Figure that illustrates Participant flow

GV, gastric varices; EV, esophageal varices; EBL, endoscopic band; EVO, endoscopic variceal obturation.
